# Podocalyxin-like and RNA-binding motif protein 3 are prognostic biomarkers in urothelial bladder cancer: a validatory study

**DOI:** 10.1186/s40364-017-0090-y

**Published:** 2017-03-14

**Authors:** Karolina Boman, Gustav Andersson, Christoffer Wennersten, Björn Nodin, Göran Ahlgren, Karin Jirström

**Affiliations:** 1 Department of Clinical Sciences Lund, Division of Oncology and Pathology, Lund University, Lund, Sweden; 2 Department of Translational Medicine, Lund University, Malmö, Sweden; 3 Department of Urology, Skåne University Hospital, Malmö, Sweden

**Keywords:** PODXL, RBM3, Bladder cancer, Prognosis

## Abstract

**Background:**

Urothelial bladder cancer (UBC) is a disease that often is discovered when the tumour is non-muscle invasive, i.e. in Ta or T1 stage. Some patients will progress into muscle-invasive disease, a potentially deadly condition. Although there are some prognostic models, the need for prognostic and predictive biomarkers is considerate and urgent. Membranous expression of podocalyxin-like protein 1 (PODXL) and low expression of the RNA-binding motif 3 (RBM3) has previously been shown to be associated with an aggressive tumour phenotype and poor prognosis in several forms of cancer, including UBC. In this study, we sought to validate the prognostic impact of PODXL and RBM3 in an independent cohort of UBC.

**Methods:**

Using tissue microarrays and immunohistochemistry, PODXL and RBM3 expression was evaluated in 272 incident UBC cases from the prospective, population-based cohort study Malmö Diet and Cancer. Kaplan-Meier analysis and Cox proportional hazards modelling were used to evaluate the prognostic impact of these markers on 5-year overall survival (OS).

**Results:**

In line with previous studies, both membranous PODXL expression and low RBM3 expression was significantly associated with disadvantageous clinicopathological features. Membranous PODXL expression was significantly associated with a reduced 5-year overall survival in the entire cohort (univariable HR 3.28; 95% CI 1.89–5.69), but this association did not remain significant in multivariable analysis. In T1 tumours, PODXL was significantly associated with reduced survival in univariable analysis (HR = 2.83; 95% CI 1.04–7.72) and borderline significant in multivariable analysis (HR = 2.60; 95% CI 0.91–7.39). Low RBM3 expression was an independent predictor of a reduced survival in the entire cohort (univariable HR 3.19; 95% CI 2.02–5.04, and multivariable HR 1.85; 95% CI 1.11–3.09), and in T1 tumours (univariable HR 2.64; 95% CI 1.11–6.27, and multivariable HR 2.63; 95% CI 1.01–6.84).

**Conclusions:**

A link between membranous PODXL expression and clinically more aggressive tumours was further confirmed, but PODXL expression was not an independent prognostic biomarker in this study. Low RBM3 expression was validated as an independent factor of poor prognosis in UBC, including T1 disease. These findings suggest that these biomarkers could be useful in stratifying patients with non-muscle invasive disease for more aggressive first line treatment.

**Electronic supplementary material:**

The online version of this article (doi:10.1186/s40364-017-0090-y) contains supplementary material, which is available to authorized users.

## Background

The majority of patients with urothelial bladder cancer (UBC) have Ta and T1 tumours, i.e. non-muscle invasive (NMI) disease, at diagnosis [1]. However, the unpredictable behaviour of these tumours regarding recurrence and progression into muscle-invasive disease makes treatment decisions difficult. NMI lesions are typically managed with transurethral resection, and selectively with intravesical therapy [2]. Patients with T1 disease, particularly those with high-risk features, are at risk of disease progression and may benefit from additional therapy. In a recent study of a large cohort of patients with non-muscle invasive UBC, the progression rates in T1G3 disease were around 20%, despite local treatment with Bacillus Calmette Guerin (BCG) [3]. Patients who progress into muscle-invasive disease (T2–T4) have a 50% risk of developing distant metastasis, even with potentially curative surgery [4]. Moreover, patients with T1 disease that progresses into MI disease seem to have a worse prognosis than those who present as primary MI [5, 6]. Surgery via radical cystectomy and pelvic lymph node dissection, with or without neoadjuvant therapy, remain the gold standard for potentially curative T2–T4 tumours. The survival rates 5 years after surgery span between 25 and 80%, being worse with higher pT stage and the presence of lymph node metastasis [4].

Hence, there is a vast and urgent need to find predictive biomarkers to help clinicians identify the patients with non-invasive tumours in need of more aggressive first line treatment, since accurate prediction of the risk of progression into muscle-invasive disease could be the key to improving patient survival in the non-muscle invasive tumour group.

Podocalyxin-like protein 1 (PODXL) is a protein involved in cell adhesion and morphology [7, 8]. Strong, in particular membranous, expression has been found to correlate with more aggressive tumours and poor survival in many cancer forms such as breast cancer [9], colorectal cancer [10–12], ovarian cancer [13], glioblastoma [14] as well as UBC [15]. In the latter study, we showed that PODXL is an independent risk factor for progressive disease and death in patients with all T-stages of UBC, as well as in the Ta/T1 subgroup.

RNA-binding motif protein 3 (RBM3) is an RNA and DNA binding protein that has previously been shown to be upregulated in cancer tissues compared with normal tissue [16], but to be associated with favourable prognosis in several major cancer forms such as breast, ovarian, prostate, testicular, esophageal, colorectal cancer and malignant melanoma [17–25]. In a previous study, we examined the prognostic value of RBM3 expression in a large group of patients with UBC (*n* = 343), and found reduced expression to be associated with clinically more aggressive tumours and an independent factor of poor prognosis in the cohort as a whole, as well as in the Ta/T1 group [26].

The aim of this study was to validate the clinicopathological correlates and prognostic impact of PODXL and RBM3 expression, respectively, in an independent cohort of UBC.

## Methods

### Study group

The study encompasses tumours from incident cases of UBC in the Malmö Diet and Cancer study (MDCS). The MDCS is a prospective population-based study, primarily aimed at examining the impact of a Western diet, low in fruit and vegetables and high in fat, on the risk of certain types of cancer. The baseline examinations took place between 1991 and 1996 in Malmö, Sweden. The source population invited to participate was comprised by women born between 1923 and 1950 and men born between 1923 and 1945 (74,138 persons). The cohort and the recruitment procedures have been published elsewhere [27]. Participants completed a detailed questionnaire, anthropometric measurements and a dietary assessment. A total of 28,098 participants completed all baseline examinations. The Ethical Committee at Lund University approved the MDCS. At the end of follow-up, December 31 2010, a total of 355 cases of incident urothelial cancer (UC) were recorded, of which 335 (95,7%) were located to the bladder. All cases of UC were histopathologically re-evaluated by a board certified pathologist according to the WHO grading system of 2004. The cohort has been described in detail previously [28] The mean and median follow-up time was 6.25 and 5.04 years, respectively (range 0.11–20.69).

### TMA construction and immunohistochemistry

Tissue microarrays (TMAs) were constructed using a semi-automated arraying device (TMArrayer, Pathology Devices, Westminster, MD, USA). All tumour samples were represented in duplicate tissue cores of 1 mm. Staining of both RBM3 and PODXL was evaluated by two independent observers (KJ and KB) who were blinded to clinical and outcome data. Scoring differences were discussed in order to reach consensus. A total number of 272 tumours, of which 264 (97.1%) were located to the bladder, were eligible for TMA construction. The cases excluded from TMA-construction had either only cytology or autopsy specimens available, or an insufficient amount of tumour tissue in the biopsies. A comparison of the TMA cohort and non-TMA cohort is provided in [28].

For immunohistochemical analysis, 4-μm TMA sections were automatically pre-treated using the PT Link system and then stained in an Autostainer Plus (Dako, Glostrup, Denmark). For evaluation of PODXL expression, the antibody AMAb90667, Atlas Antibodies AB, Stockholm, Sweden, diluted 1:250, was used. The expression of PODXL was recorded as negative (0), weak cytoplasmic positivity in any proportion of cells (1), moderate cytoplasmic positivity in any proportion (2), distinct membranous positivity in ≤50% of cells (3) and distinct membranous positivity in >50% of cells, as previously described [10, 11, 15].

For evaluation of RBM3 expression, the mouse monoclonal anti-RBM3 antibody AMAb90655, Atlas Antibodies AB, Stockholm, Sweden, diluted 1:150 was used. RBM3 expression was mainly expressed in the nuclei and the fraction of cells with nuclear positivity (NF) denoted as 0 (0–1%), 1 (2–25%), 2 (26–75%) and 3 (>75%), and the intensity of the staining ((NI) as 0 (negative), 1 (moderate) and 2 (strong). A combined nuclear score (NS), e.g. multiplier of NF x NI, was then constructed as previously described [18, 19, 26].

### Statistical methods

Chi-square test was used for comparison of biomarker expression with patient and tumour characteristics. Kaplan-Meier analysis and log-rank test were applied to illustrate differences in survival between different strata of RBM3 and PODXL expression. Cox regression proportional hazards models were used to estimate the impact of RBM3 and PODXL expression on 5-year overall survival (OS) in both univariable and multivariable analysis, adjusted for age at diagnosis (continuous), sex, T-stage and tumour grade. Classification and regression tree (CRT) analysis was applied to find an appropriate cut off value for RBM3 expression.

All calculations were performed using IBM SPSS Statistics for Mac version 23.0 (IBM, Armonk, NY, USA). All statistical tests were two-sided and a *P*-value <0.05 was considered statistically significant.

## Results

### Distribution of PODXL and RBM3 expression

After antibody staining and optimization, PODXL expression could be evaluated in 262/264 (99.2%) tumours. Membranous PODXL staining was denoted in 27/262 cases (10.3%) and there was no obvious heterogeneity between duplicate cores.

RBM3 expression could be evaluated in 259/264 (98.1%) tumours, whereby negative RBM3 staining (NS = 0) was denoted in 26 (10.0%) cases, intermediate staining in 78 (30.1%) cases and strong staining in >75% of the cells in 155 (59.8%) cases. Interestingly, all cases with NS 6 or 9, i.e. tumours denoted as being RBM3 high, were homogenously so. The tumours with lower expression (NS 0–4) had some heterogeneity between cores, but remained in the low RBM3 category. The “worst nuclear score” from each tumour was chosen for further analysis.

**Fig. 1:**
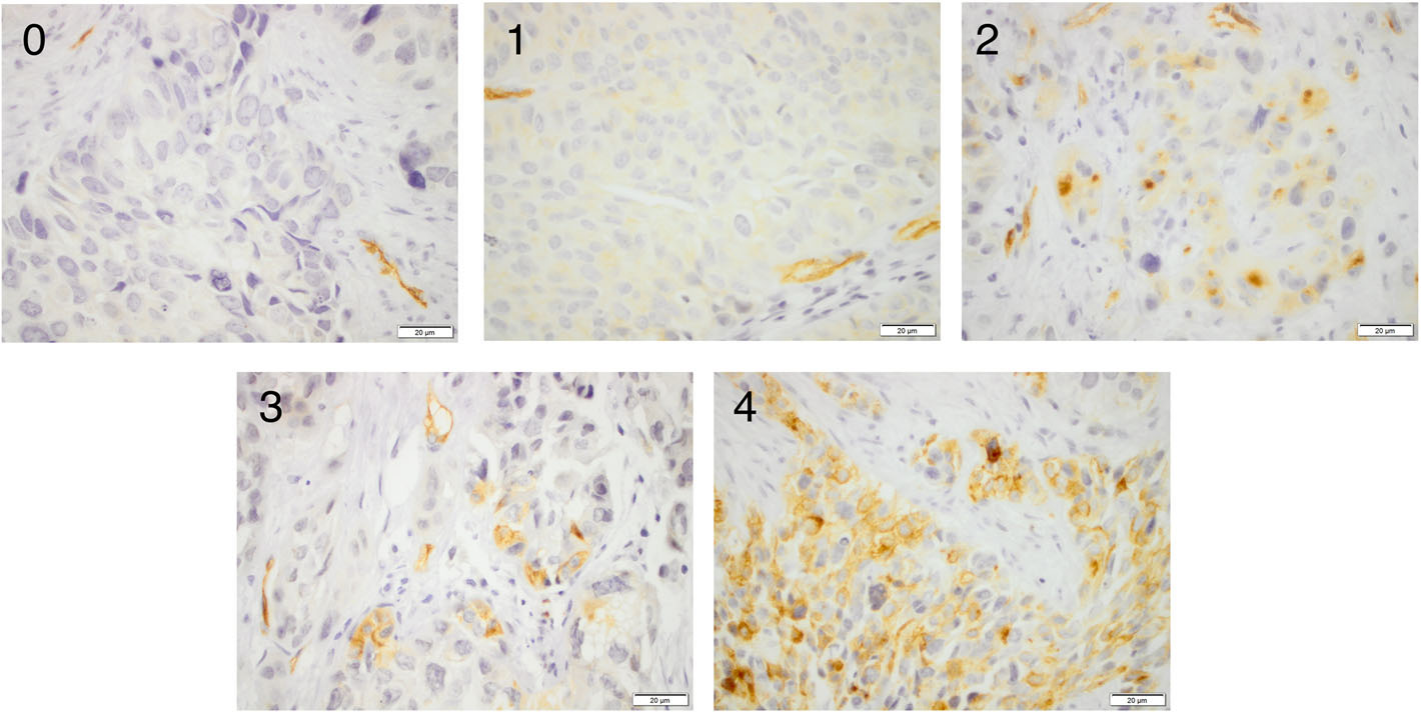
Sample immunohistochemical images of PODXL staining. Images (40× magnification) representing all different scores from 0 to 4, with 3 and 4 denoting membranous expression in < =50% or > 50% of tumour cells, respectively. Of note, score 2 represents a strong “dot-like” cytoplasming staining

**Fig. 2:**
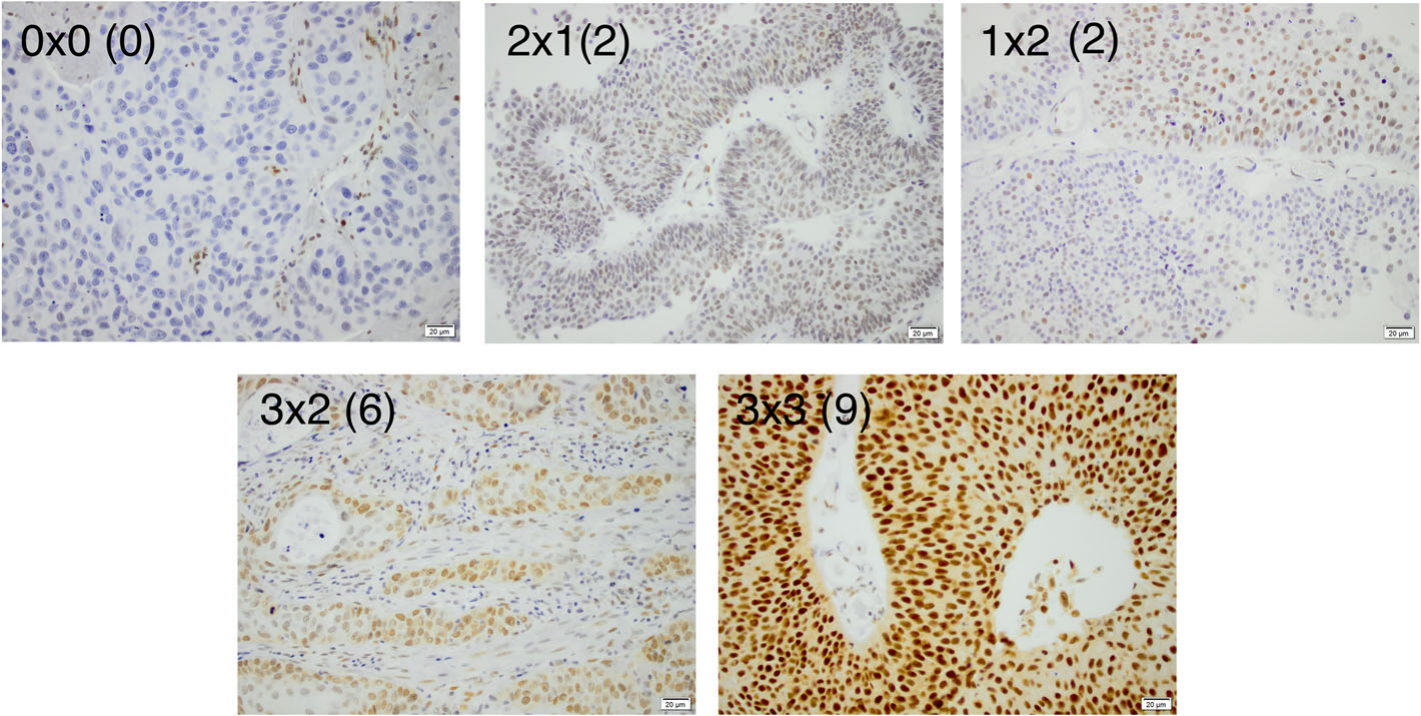
Sample immunohistochemical images of RBM3 staining. Images (20× magnification) representing different nuclear scores of RBM3 staining, i.e. fraction × intensity

Sample images of the different PODXL and RBM3 stainings are shown in Figs. 1 and 2, respectively.

### Associations of PODXL and RBM3 expression with clinicopathological factors

The associations of PODXL and RBM3 expression, respectively, with patient and tumour characteristics are shown in Table 1.

**Table 1:** Associations between RBM3 and PODXL expression and clinicopathological characteristics

PODXL Expression (*n* = 262)				RBM3 expression (*n* = 259)		
	Non-membranous	Membranous		Low (NS = 0–4)	High (NS = 6–9)	
N (%)	235 (89.7%)	27 (10.3%)	*p-value*	104 (40.2%)	155 (59.8%)	*p-value*
Age						
Mean/median	71.4/71.4	71.2/72.4	0.933	72.2/72.5	70.6/70.5	0.069
Range	51.2–86.6	56.2–86.8		51.3–86.8	51.2–86.6	
Sex						
Female	67 (84.8%)	12 (15.2%)	0.088	32 (40.0%)	48 (60.0%)	0.973
Male	168 (91.8%)	15 (8.2%)		72 (40.2%)	107 (59.8%)	
T-stage						
Ta	113 (100.0%)	0 (0.0%)	<0.001	30 (26.3%)	84 (73.7%)	<0.001
T1	73 (88.0%)	10 (12.0%)		28 (34.6%)	53 (65.4%)	
T2–4	49 (74.2%)	17 (25.8%)		46 (71.9%)	18 (28.1%)	
Grade						
Low	128 (99.2%)	1 (0.8%)	<0.001	27 (20.8%)	103 (79.2%)	<0.001
High	107 (80.5%)	26 (19.5%)		77 (59.7%)	52 (40.3%)	

Membranous PODXL expression was significantly associated with higher T-stage and high-grade tumours (*p* < 0.001 for both). High RBM3 expression was strongly and significantly associated with lower T-stage and low-grade tumours (*p* = 0.001 for both).

None of the investigated biomarkers were significantly associated with sex, or age at diagnosis.

### Association between PODXL expression and survival

The expression of PODXL was dichotomized into membranous and non-membranous expression in line with previous studies [11, 15].

**Fig. 3:**
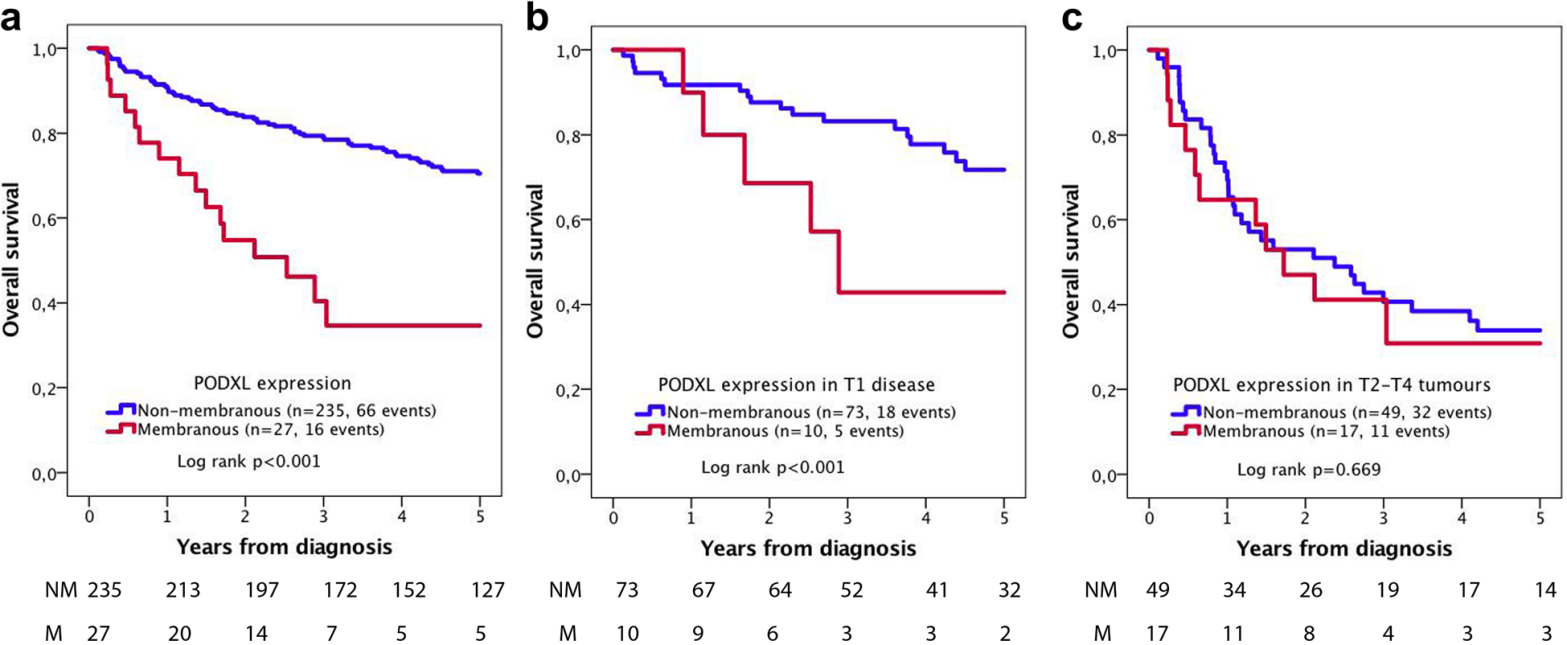
Five-year overall survival according to PODXL expression. Kaplan-Meier analysis of PODXL expression in relation to 5-year overall survival in (**a**) the full cohort, (**b**) T1 tumours and (**c**) T2–T4 tumours

As shown in Fig. 3, Kaplan-Meier analysis of the entire cohort demonstrated a significant association between membranous PODXL and a shorter 5-year OS in the entire cohort (*p* < 0.001, Fig. 3a), and in T1 disease (*p* < 0.001, Fig. 3b). PODXL was not prognostic in T2–T4 disease (Fig. 3c). Notably, no membranous PODXL expression was seen in Ta tumours.

As shown in Table 2, univariable Cox regression analysis confirmed the significant association between membranous PODXL expression and a reduced 5-year OS in the entire cohort (HR 3.28; 95% CI 1.89–5.69) and in T1 tumours (HR 2.83; 95% CI 1.04–7.72). These associations did however not remain significant in multivariable analysis (Table 2).

**Table 2:** Relative risk of overall death within 5 years according to clinicopathological factors and expression of PODXL and RBM3

	n (events)	Univariable	*P-value*	Multivariable	*P-value*
		HR (95% CI)		HR (95% CI)	
Entire cohort					
Age^a^					
Continuous	258 (79)	1.04 (1.01–1.07)	*0.010*	0.96 (0.59–1.57)	*0.875*
Sex^a^					
Female	79 (23)	1.00		1.00	
Male	179 (56)	1.06 (0.65–1.72)	*0.827*	1.03 (1.00–1.06)	*0.078*
Grade^a^					
Low	129 (20)	1.00		1.00	
High	129 (59)	3.86 (2.32–6.42)	*<0.001*	1.61 (0.82–3.18)	*0.165*
Stage^a^					
Ta	113 (16)	1.00		1.00	
T1	81 (21)	2.20 (1.14–4.21)	*0.018*	1.69 (0.81–3.56)	*0.165*
T2–T4	64 (42)	8.20 (4.58–14.66)	*<0.001*	4.85 (2.23–10.55)	*<0.001*
PODXL expression					
Non-membranous	235 (66)	1.00		1.00	
Membranous	27 (16)	3.28 (1.89–5.69)	*<0.001*	1.49 (0.83–2.68)	*0.181*
RBM3 expression					
High	155 (29)	1.00		1.00	
Low	104 (50)	3.19 (2.02–5.04)	*<0.001*	1.85 (1.11–3.09)	*0.018*
T1 disease					
Age^a^					
Continuous	81 (21)	1.01 (0.96–1.07)	*0.604*	1.08 (1.00–1.17)	*0.066*
Sex^a^					
Female	21 (6)	1.00		1.00	
Male	60 (15)	1.05 (0.41–2.72)	*0.914*	0.93 (0.36–2.42)	*0.884*
Grade^a^					
Low	28 (4)	1.00		1.00	
High	53 (17)	2.17 (0.73–6.47)	*0.163*	1.63 (0.50–6.84)	*0.412*
PODXL expression					
Non-membranous	73 (18)	1.00		1.00	
Membranous	10 (5)	2.83 (1.04–7.72)	*0.042*	2.60 (0.91–7.39)	*0.073*
RBM3 expression					
High	53 (9)	1.00		1.00	
Low	28 (12)	2.64 (1.11–6.27)	*0.028*	2.63 (1.01–6.84)	0.047
T2–T4 disease					
Age^a^					
Continuous	64 (42)	1.00 (0.96–1.04)	*0.935*	1.08 (1.00–1.17)	*0.066*
Sex^a^					
Female	18 (12)	1.00		1.00	
Male	46 (30)	0.77 (0.39–1.52)	*0.453*	0.93 (0.36–2.42)	*0.884*
Grade^a^					
Low	4 (2)	1.00		1.00	
High	60 (40)	1.79 (0.43–7.44)	*0.424*	1.63 (0.51–5.25)	*0.412*
PODXL expression					
Non-membranous	47 (31)	1.00		1.00	
Membranous	17 (11)	1.16 (0.54–2.31)	*0.669*	1.08 (0.53–2.20)	*0.832*
RBM3 expression					
High	18 (9)	1.00		1.00	
Low	46 (33)	2.01 (0.95–4.23)	*0.066*	2.22 (0.94–5.28)	*0.071*

^a^Cases included in the univariable analysis of clinicopathological factors were those that had information on both PODXL and RBM3 expression

### Association between RBM3 expression and survival

Kaplan-Meier analysis of different categories of RBM3 expression (NS) revealed a stepwise reduced 5 year overall survival (OS), with the shortest survival for RBM3 negative tumours and the longest survival for the two highest categories, i.e. NS 6 and 9 (Additional file 1). CRT analysis confirmed these associations (Additional file 2). Hence, for statistical purposes, categories of the expression of RBM3 were dichotomized into low (NS = 0–4), and high (NS = 6–9).

**Fig. 4:**
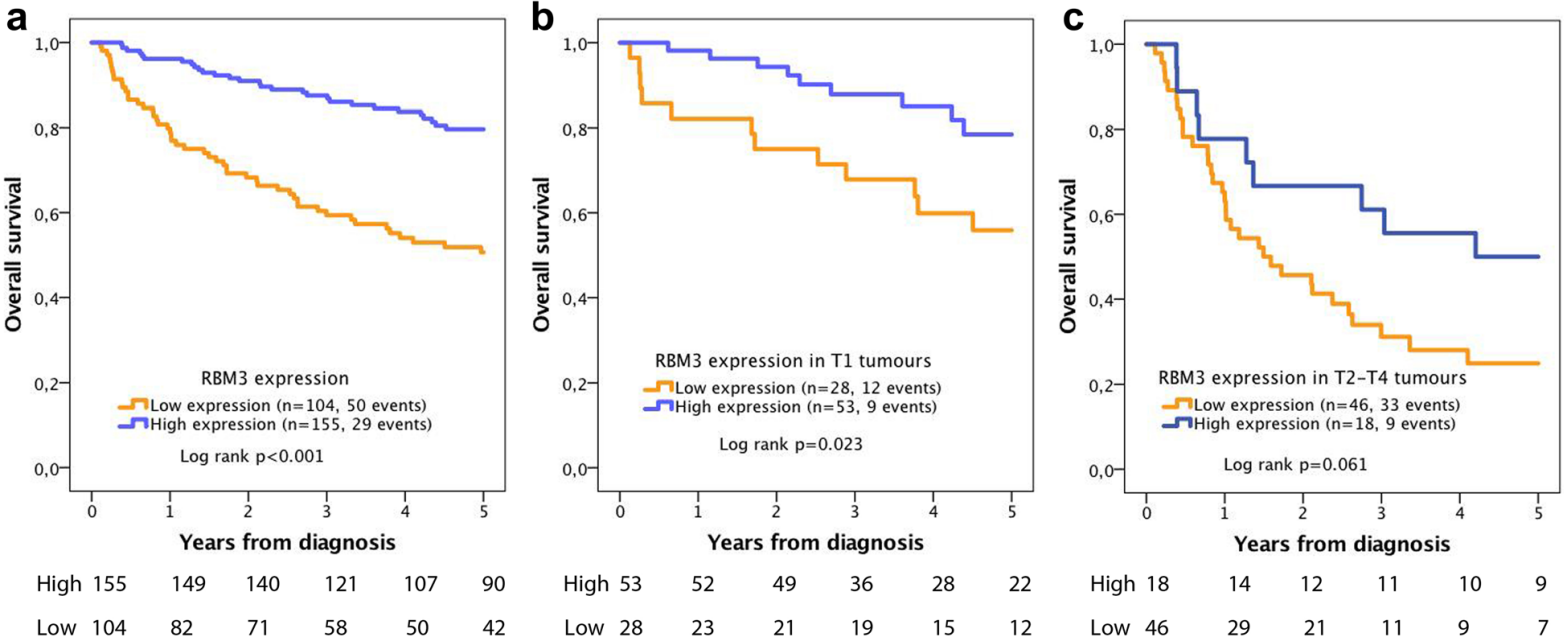
Five-year overall survival according to RBM3 expression. Kaplan-Meier analysis of RBM3 expression in relation to 5-year overall survival in (**a**) the full cohort, (**b**) T1 tumours and (**c**) T2–T4 tumours

Kaplan Meier analysis revealed that cases with low RBM3 expression had a significantly shorter 5-year OS than cases with high expression (*p* < 0.001, Fig. 4a). There was no significant association between RBM3 expression and survival in Ta tumours (data not shown), but in T1 tumours, low RBM3 expression was significantly associated with a reduced 5-year OS (*p* = 0.023, Fig. 4b). In muscle-invasive (T2–T4) tumours, there was a borderline significant trend towards a reduced 5-year OS for tumours with low RBM3 expression (*p* = 0.061, Fig. 4c).

As shown in Table 2, univariable Cox regression analysis confirmed the significant association between low RBM3 expression and a shorter 5-year OS in the entire cohort (HR 3.19; 95% CI 2.02–5.02) and in T1 tumours (HR 2.64; 95% CI 1.11–6.27). In multivariable analysis, adjusted for age, sex, T-stage and grade, RBM3 remained an independent prognostic factor in the entire cohort (HR 1.85; 95% CI 1.11–3.09) and in T1 tumours (HR 2.63 95% CI 1.01–6.84). In T2–T4 tumours, none of the established clinicopathological factors remained significant, but RBM3 was borderline prognostic in both univariable (HR 2.01, 95% CI 0.95–4.23) and multivariable analysis (HR 2.22, 95% CI 0.94–5.28).

When both PODXL and RBM3 were included in the multivariable model, only low RBM3 expression and T-stage remained significant prognostic factors in the entire cohort (HR 1.97; 95% CI 1.18–3.31 and HR 4.88; 95% CI 2.15–11.09, respectively). In the T1 subgroup, only RBM3 showed a significant association with survival (HR 2.79; 95% CI 1.05–7.36). In the T2–T4 group, none of the investigated biomarkers remained significant, although RBM3 was borderline so (HR 2.35; 95% CI 0.97–5.72).

### PODXL and RBM3 expression in relation to tumour type

The re-classified tumours were also divided into four categories according to tumour type, i.e. non-invasive low grade (*n* = 98), non-invasive high grade (*n* = 16), classic invasive (*n* = 99) and invasive non-classic (*n* = 51). The non-classic tumour group included UBC with giant cell carcinoma (*n* = 2), nested (*n* = 1), glandular (*n* = 5), squamous (*n* = 14), trophoblastic (*n* = 2), microcystic (*n* = 3), micropapillary (*n* = 9), plasmacytoid (*n* = 1) and sarcomatoid differentiation (*n* = 11). Kaplan-Meier analysis demonstrated that the 5-year OS differed significantly between groups, with the lowest 5-year OS for the classic group (Additional file 3). This was confirmed in univariable Cox regression analysis, with the non-classic tumour group having a significantly reduced survival (HR 8.58, 95% CI 4.87–15.12, *p* < 0.001) and this association remained significant in multivariable analysis (HR 5.87, 95%CI 1.98–17.44, *p* = 0.001). As shown in Table 3, non-classic tumour types were significantly correlated with a more advanced higher T-stage (*p* < 0.001), grade (*p* < 0.001), membranous PODXL expression (*p* < 0.001) and low RBM3 expression (*p* < 0.001).

**Table 3:** Associations between RBM3 and PODXL expression and tumour type

PODXL expression				RBM3 expression			
	Non-membranous	Membranous			Low (NS = 0–4)	High (NS = 6–9)	
Tumour type				Tumour type			
Non-invasive low grade (*n* = 97)	97 (100%)	0 (0%)	*p < 0.001*	Non-invasive low grade (*n* = 98)	25 (25.5%)	73 (74.5%)	*p < 0.001*
Non-invasive high grade (*n* = 16)	16 (100%)	0 (0%)		Non-invasive high grade (*n* = 16)	5 (31.3%)	11 (68.7%)	
Invasive classic UBC (*n* = 99)	85 (85.9%)	14 (14.1%)		Invasive classic UBC (*n* = 99)	41 (42,3%)	54 (57.7%)	
Invasive non-classic UBC (*n* = 50)	37 (74.0%)	13 (26.0%)		Invasive non-classic UBC (*n* = 48)	33 (68.8%)	15 (31.2%)	

After adding tumour type as a factor into the multivariable Cox regression analysis of the entire cohort, together with both PODXL and RBM3, RBM3 expression remained significant (HR 1.84, 95% CI 1.09–3.10, *p* = 0.022), but not PODXL expression (HR 1.43, 95% CI 0.77–2.67, *p* = 0.257). Addition of tumour type to the multivariable analyses in Table 2 yielded similar results (data not shown).

## Discussion

This study examined the prognostic impact of PODXL and RBM3 expression in tumours from incident cases of UBC in a large, population-based cohort. The results provide further evidence of low RBM3 expression being associated with unfavourable clinicopathological characteristics and an independent factor of decreased survival, and of PODXL expression being associated with more aggressive tumour characteristics and reduced survival, however not independently of established prognostic factors.

PODXL is a protein that in experimental studies is associated with epithelial mesenchymal transition (EMT) and, hence, the invasion and spread of tumours [29]. High expression of PODXL, in particular in the membrane of tumour cells, has previously been demonstrated to correlate to an impaired prognosis in many major cancer forms like breast cancer [9], colorectal cancer [10–12], ovarian cancer [13], glioblastoma [14] and urothelial bladder cancer [15]. The results from this study further validate that membranous expression of PODXL is correlated to more aggressive tumours as it could not be demonstrated in non-invasive Ta-tumours. In addition, it was significantly associated with a reduced survival in univariable analysis, in the full cohort and in T1 disease, but not in T2–T4 disease. The latter may perhaps reflect the fact that more aggressive and unstable tumours reside in the MI group, therefore diluting the prognostic impact of PODXL expression in the NMI group. Although this study failed to demonstrate a significant association between PODXL expression and survival in multivariable analysis, it should be pointed out that, apart from T-stage, that was an independent prognostic factor in the entire cohort, no other factors provided an independent prognostic value. PODXL expression was borderline significantly associated with a reduced survival in multivariable analysis in the T1 group, which is in line with previous findings [15]. Hence, the prognostic value of PODXL appears to be most evident in T1 disease, and its expression may possibly add value to current prognostic models.

Herein, a significantly decreased 5-year OS was demonstrated for patients with tumours displaying low expression of RBM3, which is well in line with previous findings in UBC [26], as well as breast cancer [17], epithelial ovarian cancer [18, 30], prostate cancer [19, 21], testicular cancer [25], oesophageal and gastric cancer [23] and melanoma [24].

On a cellular level, RBM3 is normally expressed when cells are exposed to conditions such as hypoxia and cell starvation. These conditions are present in the environment of a malignant tumour [31]. The role of RBM3 in adverse growth conditions is to increase cell survival by means of hindering the normal decrease of protein synthesis and promoting cells to continue through the cell cycle [16, 32]. It has the making of a powerful oncoprotein, but is linked to an improved survival. Perhaps this reflects the regulatory role of its binding to RNA and modulation of RNA expression, making cells more genetically stable, or to the correlation between downregulation of RBM3 and reduced sensitivity to cisplatin in epithelial ovarian cancer in vitro [18], presumably by processes involved in DNA integrity and repair [30]. The indication that RBM3 may be a predictive marker for cisplatin response is in line with the observed association between low RBM3 expression and treatment failure in non-seminatous testicular germ cell cancer [25], in which cisplatin treatment is highly effective. Cisplatin is frequently used for neoadjuvant treatment in MI and palliative treatment in metastatic UBC [4]. Therefore, it would be of interest to study the association of RBM3 with cisplatin sensitivity in this type of cancer.

The T1 category is a subgroup with an urgent need of prognostic biomarkers, as they have a non-negligible risk for progression to muscle-invasive stages [33]. Interestingly, low RBM3 expression was the only significant independent marker of poor prognosis in the T1 group. Therefore, RBM3 may be a clinically useful marker to select patients with high risk of muscle-invasive disease, and, hence, in need of early cystectomy.

Non-classical differentiation was associated with low RBM3 expression, membranous PODXL expression and an adverse outcome for patients. The tumour types included in this group are known to have a poor prognosis compared to classic UBC, apart from microcystic differentiation, for which the clinical relevance is unknown [34, 35]. The association of non-classical tumours with poor prognosis was validated in our study, however, these subtypes are rare and, compared to the prognostic impact of the two herein examined biomarkers, of limited clinical value to the UBC patient population as a whole.

The herein used antibody for detection of RBM expression has been extensively validated [20, 30] and the only seeming contradiction to our findings regarding the prognostic value of RBM3 expression in UBC is a TMA study on cystectomy specimens [36]. However, clinical follow-up was only available for 106 patients out of a total reported number of 274 cases reported in this study, with a mean duration of 2 years. The data presented did not meet the reporting recommendations for tumour marker prognostic studies (REMARK) criteria [37] regarding neither patient characterization, the reporting of data, nor the analysis and presentation. The difference in results between this study and ours may also be due to the fact that the most evident prognostic value of RBM3 expression seems to be for the NMI tumours. In terms of clinical application, this is certainly beneficial. The treatment of MI tumours is cystectomy if the patient is fit for surgery, but the difficulty in choosing therapies lies more in the NMI group.

## Conclusions

The results from this study validate the prognostic value of PODXL and RBM3 expression in UBC, with RBM3 being the potentially most clinically useful biomarker for prognostic stratification of patients with T1 disease. These findings merit further study and validation also in a prospective setting.

## Additional files

Additional file 1: Kaplan-Meier analysis of 5-year overall survival according to all nuclear scores of RBM3 expression. (JPG 33 kb)
Additional file 2: Specified non-classical tumour types and their relation to PODXL and RBM3 expression. (DOCX 90 kb)
Additional file 3: Kaplan-Meier analysis of 5-year overall survival according to tumour type. (JPG 764 kb)

## Abbreviations

MDCS: Malmö Diet and Cancer study
MI: Muscle invasive
NMI: Non-muscle invasive
NS: Nuclear score
PODXL: Podocalyxin like protein
RBM3: RNA-binding motif 3
TMA: Tissue Micro Array
UBC: Urothelial bladder cancer
