# Supplementary figures and images for: Podocalyxin-like and RNA-binding motif protein 3 are prognostic biomarkers in urothelial bladder cancer: a validatory study
Source: Biomark Res. 2017 Mar 14;5:10. doi: 10.1186/s40364-017-0090-y (PMC5348745; doi:10.1186/s40364-017-0090-y)

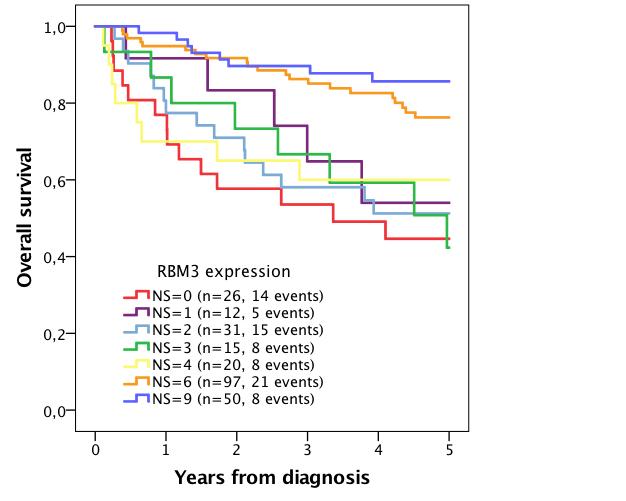

Supplement: Additional file 1: — Kaplan-Meier analysis of 5-year overall survival according to all nuclear scores of RBM3 expression. (JPG 33 kb) [file 40364_2017_90_MOESM1_ESM.jpg]

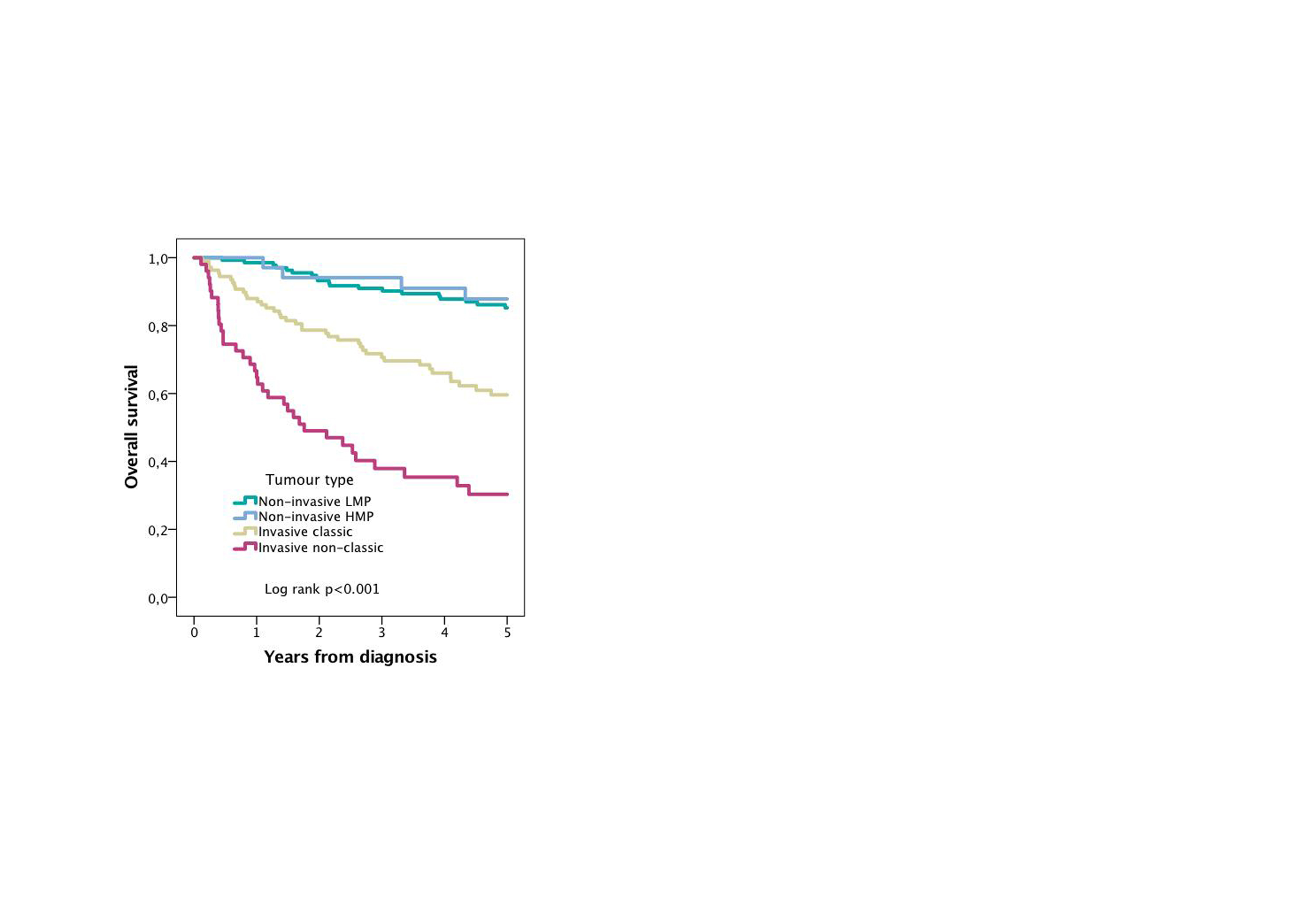

Supplement: Additional file 3: — Kaplan-Meier analysis of 5-year overall survival according to tumour type. (JPG 764 kb) [file 40364_2017_90_MOESM3_ESM.jpg]
