# Supplementary material for: Podocalyxin-like and RNA-binding motif protein 3 are prognostic biomarkers in urothelial bladder cancer: a validatory study
Source: Biomark Res. 2017 Mar 14;5:10. doi: 10.1186/s40364-017-0090-y (PMC5348745; doi:10.1186/s40364-017-0090-y)
Supplement: Additional file 2: — Specified non-classical tumour types and their relation to PODXL and RBM3 expression. (DOCX 90 kb) [file 40364_2017_90_MOESM2_ESM.docx]

| **Type of tumour** | **RBM3 expression** | |
| --- | --- | --- |
|  | *Low* | *High* |
| Infiltrating UCA NOS | N=41 | N=57 |
| Infiltrating UCA with squamous differentiation | N=10 | N=4 |
| Infiltrating UCA with glandular differentiation | N=2 | N=3 |
| Infiltrating UCA with trophoblastic differentiation | N=2 | N=0 |
| Nested UCA | N=0 | N=1 |
| Microcystic UCA | N=1 | N=2 |
| Micropapillary UCA | N=7 | N=2 |
| Plasmacytoid UCA | N=1 | N=0 |
| Sarcomatoid UCA | N=8 | N=3 |
| Giant cell UCA | N=2 | N=0 |
| Non-invasive UCA high grade | N=5 | N=11 |
| Non-invasive UCA low grade | N=25 | N=72 |

*UCA= Uroepithelial carcinoma*

| **Type of tumour** | **PODXL expression** | |
| --- | --- | --- |
|  | *Membranous* | *Non-membranous* |
| Infiltrating UCA NOS | N=14 | N=86 |
| Infiltrating UCA with squamous differentiation | N=3 | N=11 |
| Infiltrating UCA with glandular differentiation | N=2 | N=4 |
| Infiltrating UCA with trophoblastic differentiation | N=0 | N=2 |
| Nested UCA | N=0 | N=1 |
| Microcystic UCA | N=0 | N=3 |
| Micropapillary UCA | N=3 | N=6 |
| Plasmacytoid UCA | N=1 | N=0 |
| Sarcomatoid UCA | N=3 | N=9 |
| Giant cell UCA | N=1 | N=1 |
| Non-invasive UCA high grade | N=0 | N=16 |
| Non-invasive UCA low grade | N=0 | N=96 |

*UCA= Uroepithelial carcinoma*
